# IMPACT OF A 4-MINUTE STRENGTH TRAINING PROGRAM ON LOWER BODY FUNCTION IN OLDER ADULTS WITH MOBILITY DISABILITY

**DOI:** 10.1093/geroni/igad104.3410

**Published:** 2023-12-21

**Authors:** Jordan Kurth, Christopher Sciamanna, Jonathan Stine, Matthew Ladwig, David Conroy, Kathryn Schmitz, Cheyenne Herrell

**Affiliations:** The Pennsylvania State University College of Medicine, Hershey, Pennsylvania, United States; The Pennsylvania State University College of Medicine, Hershey, Pennsylvania, United States; The Pennsylvania State University College of Medicine, Hershey, Pennsylvania, United States; Purdue University Northwest, Hammond, Indiana, United States; The Pennsylvania State University College of Medicine, Hershey, Pennsylvania, United States; University of Pittsburgh, Pittsburgh, Pennsylvania, United States; The Pennsylvania State University College of Medicine, Hershey, Pennsylvania, United States

## Abstract

One in four older adults experience mobility disability, often resulting in financial strain and declines in quality of life. Though resistance training (RT) can improve physical function, too few older adults do RT. This study specifically reports on the impact of a short, daily RT program called Functional Activity Strength Training (FAST) on lower extremity function. FAST entailed one 30-second bout each of four exercises (push-ups, chair stands, two-arm resistance band rows, stair-stepping) followed by 30 seconds of rest daily for 12 weeks. Ninety-seven participants were randomly assigned to either intervention (n = 44) or delayed treatment control (n = 53) group. Research staff remotely provided one-on-one coaching relating to modifications and progress every 2 weeks until week 8. Participants received a daily email reminder to complete the exercises and report repetitions completed for each exercise. Outcome measures (5-time sit-to-stand, maximum 30 second chair stand performance, and single-leg stance duration) were evaluated at baseline and week 12. Participants completed exercises on 80% of possible days. Over the 12 weeks, the intervention group improved over and above the control group by +4.2 chair stands (d = 1.21), -2.3 seconds (d = -0.43) on the 5-times sit-to-stand, and +3.58 seconds (d = 0.46) on the single-leg stance. In sum, a four-minute daily, 12-week functional RT program led to improvements in chair stand, 5-times sit-to-stand, and single-leg stance performance. Efficacy trials, using gold-standard assessments should examine whether similar improvements are observed and adherence is maintained over a longer period of time.

